# Growth regulation by amino acid transporters in *Drosophila* larvae

**DOI:** 10.1007/s00018-020-03535-6

**Published:** 2020-05-01

**Authors:** Gérard Manière, Georges Alves, Martine Berthelot-Grosjean, Yael Grosjean

**Affiliations:** Centre des Sciences du Goût et de l’Alimentation, AgroSup Dijon, CNRS, INRA, Université Bourgogne Franche-Comté, 21000 Dijon, France

**Keywords:** Neuron, Glia, Physiology, Molecular signal, Insulin producing cells, LAT1

## Abstract

*Drosophila* larvae need to adapt their metabolism to reach a critical body size to pupate. This process needs food resources and has to be tightly adjusted to control metamorphosis timing and adult size. Nutrients such as amino acids either directly present in the food or obtained via protein digestion play key regulatory roles in controlling metabolism and growth. Amino acids act especially on two organs, the fat body and the brain, to control larval growth, body size developmental timing and pupariation. The expression of specific amino acid transporters in fat body cells, and in the brain through specific neurons and glial cells is essential to activate downstream molecular signaling pathways in response to amino acid levels. In this review, we highlight some of these specific networks dependent on amino acid diet to control DILP levels, and by consequence larval metabolism and growth.

## Introduction

To grow and to survive, animals must constantly adapt to their environment where they have to find all the essential components they need such as water, oxygen, and food. In particular, they must find in their diet all the elements necessary to cover their daily nutritional needs and to develop. To ensure an optimal supply of nutrients, animals constantly adjust their food intake to their nutritional status [1]. The ingested food is broken down during digestion into various nutrients such as fatty acids, sugars and amino acids, which are essential for energy production and cellular functioning, division, growth and renewal [2–4]. Among these, essential amino acids are key nutrients that animals cannot synthesize and need to find from their food [5]. Most amino acids also represent signaling molecules that control animal metabolism. They need to be finely regulated to assess vital requirements such as energy balance, protein synthesis, and cell and tissue development [1]. In *Drosophila*, growth occurs during four morphologically distinct developmental forms: embryo, larva (three instar stages), pupa, and adult. The most important body size increase prevails during third larval instar. This spectacular growth is concluded when a critical weight is reached, which mobilizes hormonal signals such as PTTH, ecdysone, juvenile hormone, and insulin-like peptides [6–9]. As a consequence, larvae must eat a lot during this third instar to gain weight. This review will focus on the role of amino acids transporters affecting this larval growth.

**Fig. 1:**
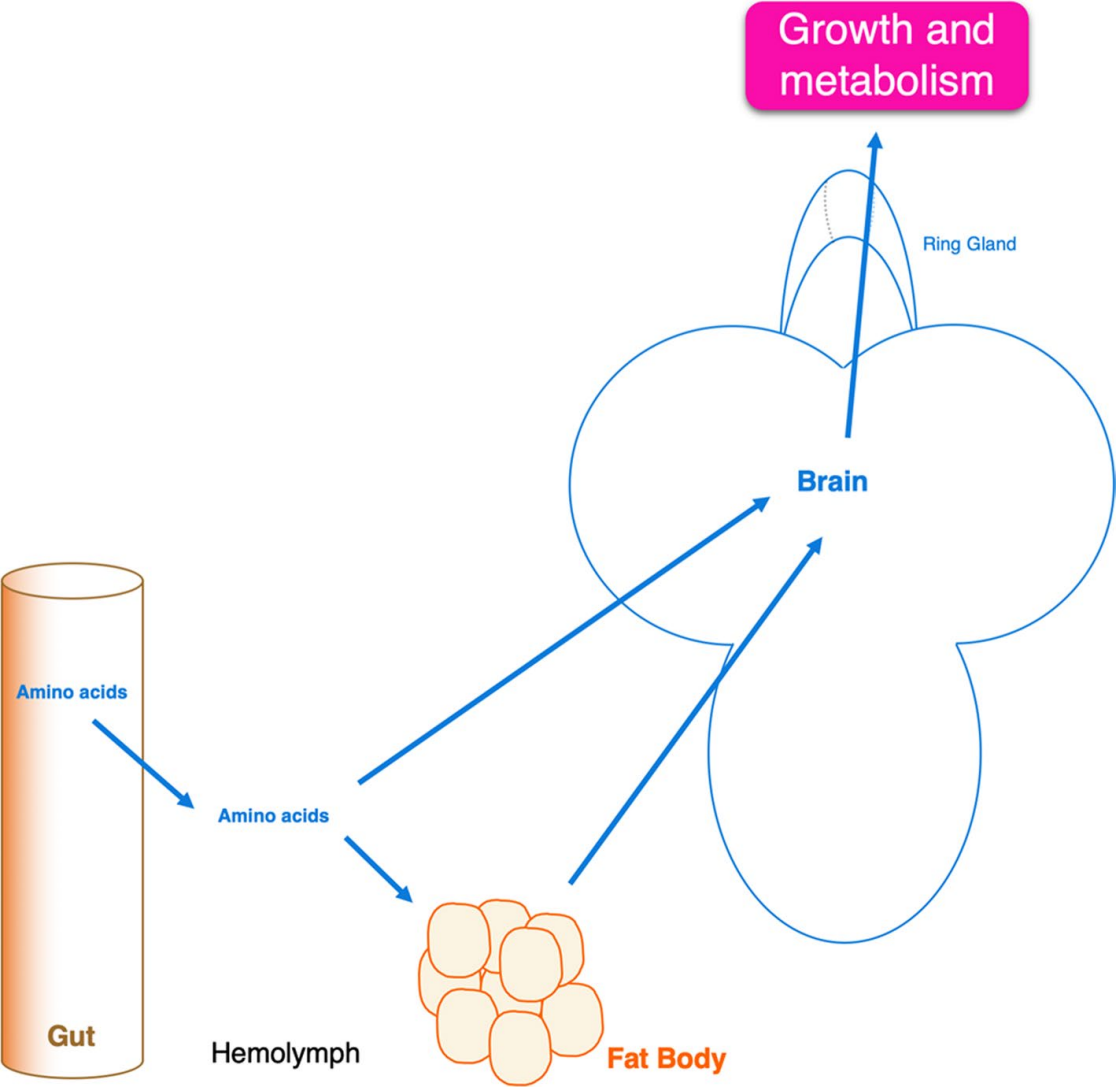
Internal amino acid sensing, and regulation of larval metabolism and growth. Amino acids and others nutrients go through the intestine wall into the hemolymph. Once in the hemolymph, these molecules can circulate in the whole body and are detected by the fat body or by the brain. Then, these two organs secrete and exchange many signaling molecules required to regulate metabolism and growth

In *Drosophila* larvae, nutrients like amino acids go across the gut wall from the lumen of the digestive tract to the hemolymph [4, 10]. Thus, amino acids circulate in the whole body before being detected and used by cells for protein synthesis or metabolic purposes. Organs that are able to detect them include the fat body, and some neurons and glial cells located in the brain. As in mammals, a permanent dialog between these tissues exists to maintain an amino acid homeostasis necessary for development and growth of *Drosophila* larvae [11]. Amino acids can, thus, modify the physiology of the organism by direct cell-autonomous, or indirect non-cell-autonomous detection (Fig. 1).

In *Drosophila* larvae, the regulation of metabolism involves many hormones including insulin-like peptides [12]. Eight insulin-like coding genes (*dilp1-8*) have been identified so far. DILPs 1–7 are thought to act through a single Drosophila insulin/IGF receptor (InR); whereas, DILP8 acts through the neuronal relaxin receptor lgr-3 [13–17]. The corresponding peptides are expressed in many tissues such as the nervous system and the digestive system [14, 15]. During the larval stage, when maggots feed, DILPs produced and secreted by the brain couple nutrient uptake with systemic growth. DILP2 and DILP5 are specifically expressed in 7 cell-pairs (IPCs, Insulin-Producing Cells) of the brain *pars intercerebralis* region [14]. These cells can be compared to the mammalian pancreatic ß cells that produce and secrete insulin [18, 19]. It has been shown that the genetic ablation of IPCs alone has a major impact on metabolism and development [20, 21]. In particular, the level of circulating sugars in the hemolymph is drastically increased when IPCs are ablated [21]. Another DILP, DILP6 is expressed in the fat body and in glial cells from the blood–brain barrier [22–25]. During post-feeding development, DILP6 plays an important role in growth regulation [22, 23].

## Amino acid sensing and secretion of hormones by the fat body

The *Drosophila* fat body is considered to be the equivalent of both vertebrate liver and adipose tissue [26]. It plays a key role in energy storage by converting fatty acids, proteins and sugars into triglycerides that are stored in lipid droplets [27]. The fat body can then mobilize these droplets when the animal needs high energy and compounds for cellular functioning or larval growth. For this purpose, the fat body is able to detect nutrient variations in the hemolymph, including amino acids. Sensing of amino acid in the fat body depends on the activity of TORC1 (target of rapamycin complex 1) signaling, which then indirectly induces the activation of the insulin pathway (InR/Pi3k) in salivary glands and imaginal discs [28]. The fat body then secretes hormonal factors, which act on different organs to control development and to adjust the metabolic status [28].

**Fig. 2:**
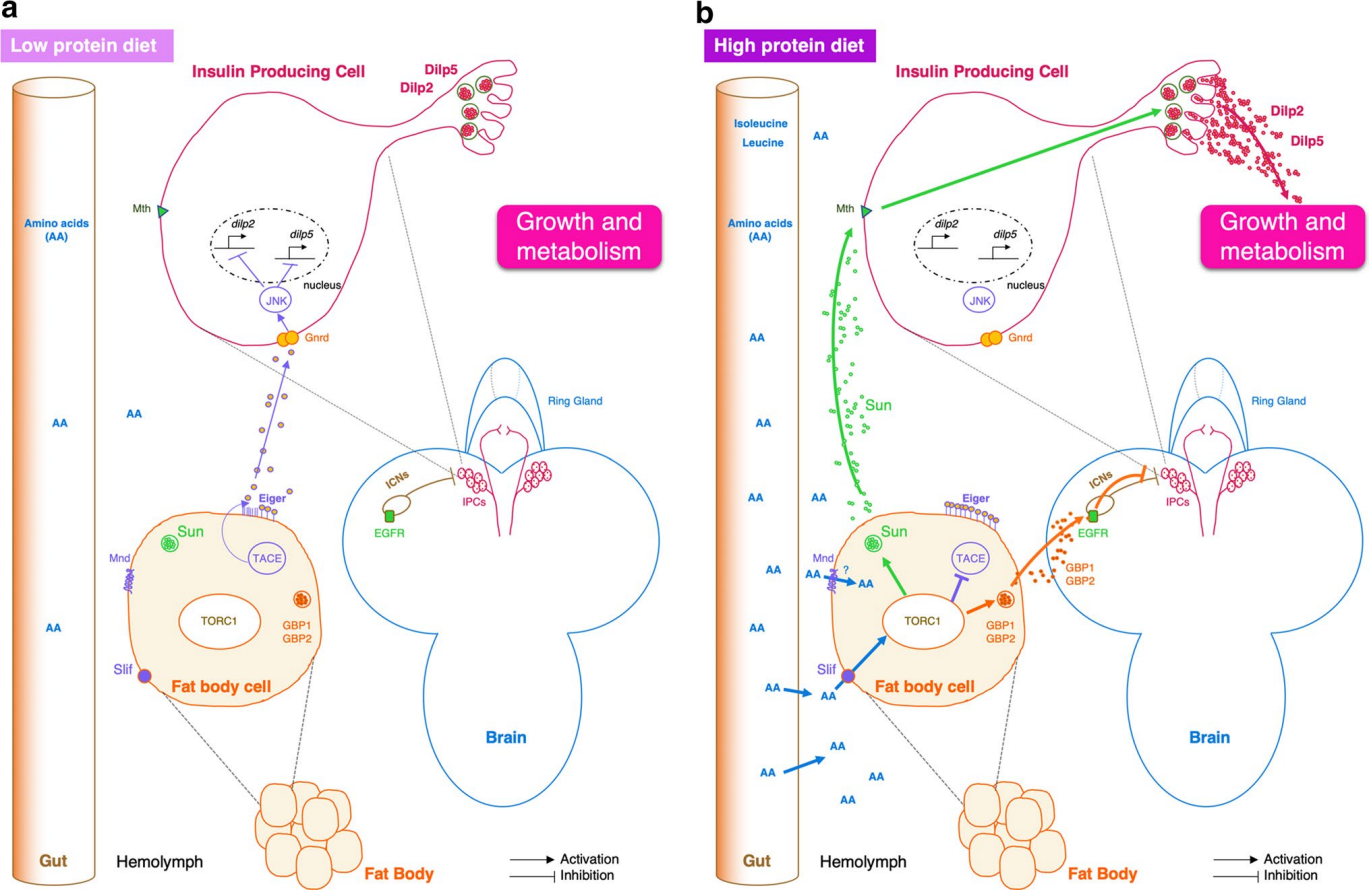
Amino acid sensing by the larval fat body. **a** In low-protein diets, the fat body secretes Eiger cytokine, which is cleaved by the enzyme TACE. Then, Eiger binds to its receptor Grindelwald (Gnrd) located on Insulin Producing Cells (IPCs) to repress expression of *dilp2* and *dilp5* genes through the Jun *N*-terminal kinase (JNK) activation. In addition, IPC-connecting neurons (ICNs) inhibit IPC neuronal activities and thus inhibit the release of DILPs. In consequence, metabolism and larval growth are reduced. **b** In high-amino acids diets, TORC1 (Target of Rapamycin) inhibits TACE and induces the release of two growth blocking peptides (GBP1 and GBP2). These peptides repress the inhibitory activity of ICNs allowing the release of DILPs and larval growth. TORC1 also promotes the secretion of Stunted (Sun) cytokine. Sun binding to its receptor Methuselah (Mth) promotes the release of DILPs, which influences metabolism and larval growth. *AA* amino acids, *DILP* Drosophila insulin-like peptide, *EGFR* EGF receptor, *GBP* growth-blocking peptide, *Gnrd* Grindelwald, *ICNs* IPC-connecting neurons, *IPCs* insulin-producing cells, *JNK* jun *N*-terminal kinase, *Mnd* minidiscs, *Mth* Methuselah, *Slif* Slimfast, *Sun* stunted, *TACE* TNFα-converting enzyme, *TORC1* target of rapamycin

The role of two amino acid transporters, Minidiscs (Mnd) and Slimfast (Slif), has been characterized in the fat body (Fig. 2). Mnd is an amino acid transporter belonging to the L-type Amino Acid Transporter (LAT1) family [29–32], which catalyzes the cross-membrane flux of large neutral amino acids. Mnd is expressed in several tissues including the fat body, and is involved in the larval development of imaginal discs [33]. *Mnd* mutant imaginal discs transplanted into a wild-type larva continue to develop. This result indicates that Mnd action on imaginal discs development is non-cell autonomous, and it suggests the existence of messengers from other organs expressing Mnd [33]. Slif belongs to the cationic amino acid family (CAT) and plays a major role in amino acid transport in the fat body, leading to TORC1 activation [34]. The inhibition of *slif* expression in the fat body causes a larval development defect that mimics an amino acid deprivation and leads to smaller adults at emergence (Fig. 2) [28].

In response to amino acids levels in the hemolymph, different factors are secreted from the fat body and induce the release of insulin-like peptides by IPCs to regulate growth. These factors have been revealed by brain and fat body co-cultures [35, 36]. Fat bodies from larvae fed with a rich amino acid medium (tryptone) are capable to stimulate the release of DILPs that are normally stored in the IPCs during starvation conditions [35]. Depending on the protein diet, two cytokines Eiger and Sun can be released by the fat body (Fig. 2a, b) [37, 38].

In low protein conditions, the fat body expresses and releases the transmembrane protein Eiger, a cytokine homologous to TNF-α in mammals [37]. Eiger is cleaved by the TNF-α converting enzyme (TACE). From this reaction, a soluble form of Eiger can circulate in the hemolymph (Fig. 2a). TACE is under the control of the TORC1 pathway that is sensitive to the presence of amino acids in the hemolymph. Eiger then binds to its receptor Grindelwald (Gnrd), a TNF-α receptor localized on the IPCs, and allows the activation of the Jun *N*-terminal Kinase (JNK) pathway. Then, activated JNK inhibits *dilp2* and *dilp5* gene expression and, therefore, blocks larval growth (Fig. 2a) [37].

In a rich protein diet, TORC1 inhibits TACE; thus, Eiger is not cleaved and remains attached to the adipocyte membrane leading to normal growth. Another cytokine, Stunted (Sun), is secreted by the fat body into the hemolymph after high amino acid diet (Fig. 2b) [38]. This release is dependent on the TORC1 pathway. Sun then binds to its receptor Methuselah, a secretin–incretin receptor expressed in IPCs. This binding permits the release of DILP2 and DILP5, which in turn activates systemic organ growth [38].

In response to a rich amino acid diet and to the activation of TORC1 pathway, the fat body secretes two other factors called GBP1 and GBP2 (Growth Blocking Peptide) that indirectly induce the release of DILP2 and DILP5 by IPCs (Fig. 2b) [36, 39]. Secreted GBP1 binds to its receptor, a transmembrane EGF receptor located on inhibitory neurons (IPC-connecting neurons or ICNs) that synapse with IPCs [39]. The binding of GBP1 to its receptor removes the inhibition of IPCs by ICNs and thus allows the release of DILPs (Fig. 2b) [39]. Thus, the intake of food or a rich amino acid diet acts through the fat body and inhibitory neurons to indirectly cause the release of DILPs by IPCs.

## Direct amino acid sensing by brain cells

**Fig. 3:**
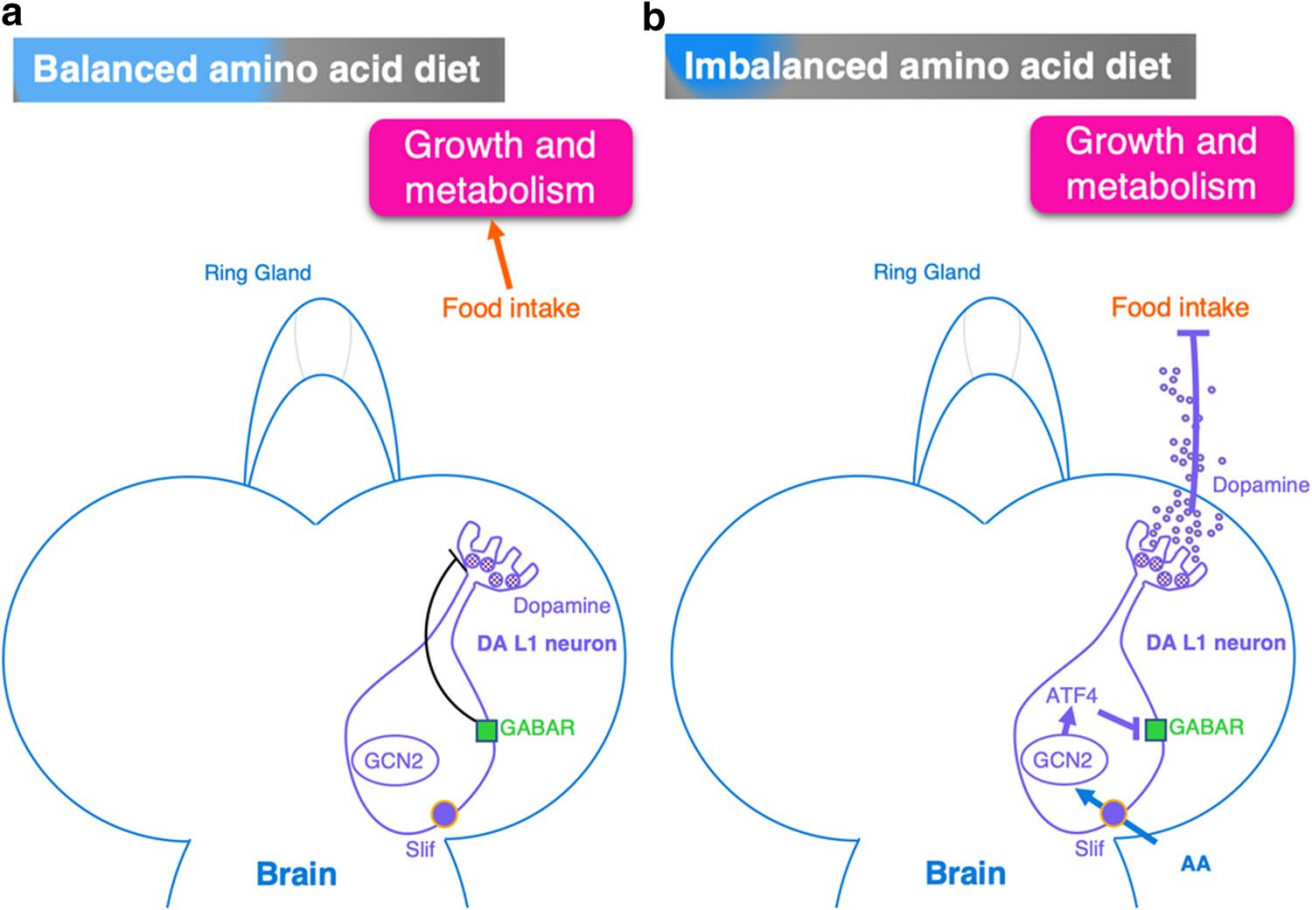
Amino acid sensing by larval dopaminergic neurons. In larva, food intake is under the control of dopaminergic neurons (DA DL1 neurons). **a** In balanced amino acid diet, the inhibition of dopamine release by GABA receptors on DA DL1 neurons allows food intake and growth. **b** In imbalanced amino acid condition, dopaminergic neurons detect amino acids transported by Slimfast (Slif) transporter. A sensor of amino acids GCN2 represses GABA receptors. The inhibition of GABA receptors promotes the release of dopamine and thus blocks food intake. *AA* amino acids, *GABAR* GABA receptor, *GCN2* GC non-derepressing 2 kinase, *GDH* glutamate dehydrogenase, *LAT-1* large neutral amino acid transporter, *Mnd* Minidiscs, *Slif* Slimfast

During the larval stages, food intake and the detection of amino acids are under the control of the nervous system allowing the larvae to distinguish balanced and imbalanced amino acid diets (Fig. 3). This control depends on a cluster of dopaminergic neurons of the dorsolateral cluster 1 (DA DL1 neurons) located in the larval brain [40]. Larvae avoid food with low essential amino acids (EAA) content and prefer food with balanced amino acids. The circulating amino acids in the hemolymph are directly detected by the DA DL1 neurons. These dopaminergic neurons have an inhibitory role in food intake since the release of dopamine reduces food consumption (Fig. 3).

In balanced amino acid diet, GABA receptors located on DA DL1 neurons repress the secretion of dopamine allowing food intake [40]. The inhibition of the expression of the amino acid transporter Slif in DA DL1 neurons reduces food intake in the presence of a rich diet (Fig. 3a) [40].

Similarly, an imbalanced amino acid diet detected by DA DL1 neurons decreases food intake (Fig. 3b). In the DA DL1 neurons, an amino acid sensor, conserved GC non-derepressing 2 (GCN2) kinase, is activated in the absence of EAA and, in turn, leads to the activation of the ATF4 transcription factor. Then, ATF4 binds to and inactivates GABAR1 and GABAR2 receptors. The inactivation of GABA receptors allows the secretion of dopamine and leads to a drop of food intake (Fig. 3b) [40].

Levels of essential amino acids can, thus, regulate food intake and, therefore, animal growth via dopaminergic DL1 neurons.

**Fig. 4:**
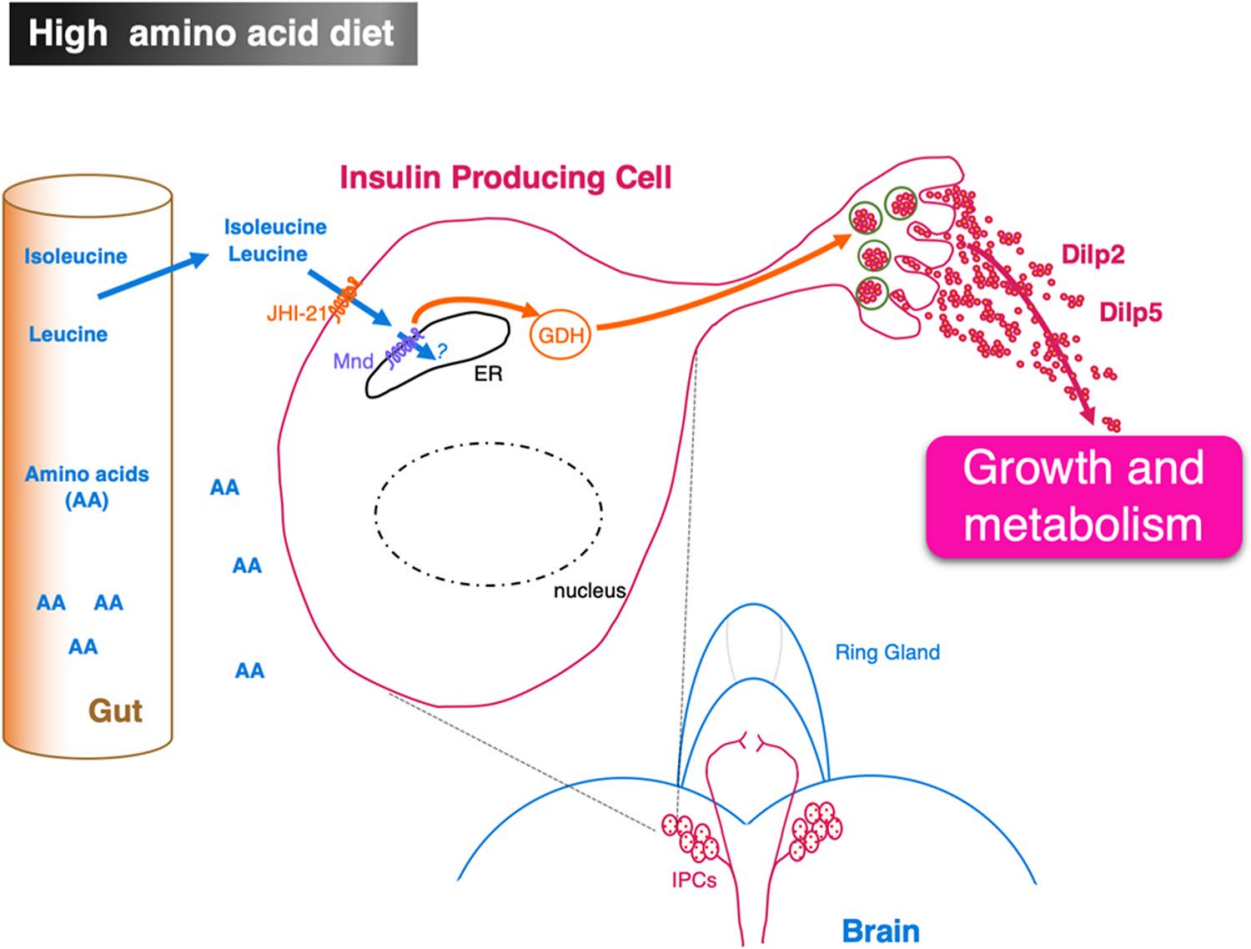
Amino acid sensing by larval insulin producing cells. Two LAT-1 transporters (Minidiscs (Mnd) and Juvenile hormone Inducible-21 (JhI-21) are expressed in IPCs. Mnd is located on the endoplasmic reticulum; whereas JhI-21 is on the plasma membrane. They allow the transport of branched amino acids (Leucine and Isoleucine) that leads to the release of DILPs. DILPs are involved in metabolism regulation and in growth control. *AA* amino acids, *ER* endoplasmic reticulum, *GDH* glutamate dehydrogenase, *LAT-1* large neutral amino acid transporter, *Mnd* Minidiscs

In mammals, amino acids such as leucine and isoleucine, which are BCAA-type amino acids (Branched Chain Amino Acids), are transported by LAT-1-type amino acid transporters and stimulate insulin release from pancreatic ß-cells [41]. In *Drosophila* larvae, homologous cells of pancreatic ß-cells are the 7 pairs of IPCs located in the median region of the *pars intercerebralis* [14, 18, 19]. In fasting *Drosophila* larvae, leucine and isoleucine are able to directly regulate the neuronal activity of these IPCs [31, 42]. Effectively, in ex vivo-cultured brains from fasting larvae, leucine induces the release of DILP2 and DILP5 by IPCs (Fig. 4). The function of two amino acid transporters of the LAT-1 family, Mnd and Juvenile hormone Inducible-21 (JhI-21), has been characterized in IPCs. Mnd is mostly present in the endoplasmic reticulum of IPCs, while JhI-21 appears to be preferentially localized at the plasma membrane of IPCs (Fig. 4) [31, 42]. Knockdown of Mnd or JhI-21 or both transporters in IPCs impairs DILPs release in the presence of leucine and has an impact on metabolism and on larval growth [31, 42]. Therefore, as in mammals, leucine stimulates a leucine sensitive enzyme GDH located in the IPCs to promote insulin release in *Drosophila* larvae (Fig. 4) [31].

**Fig. 5:**
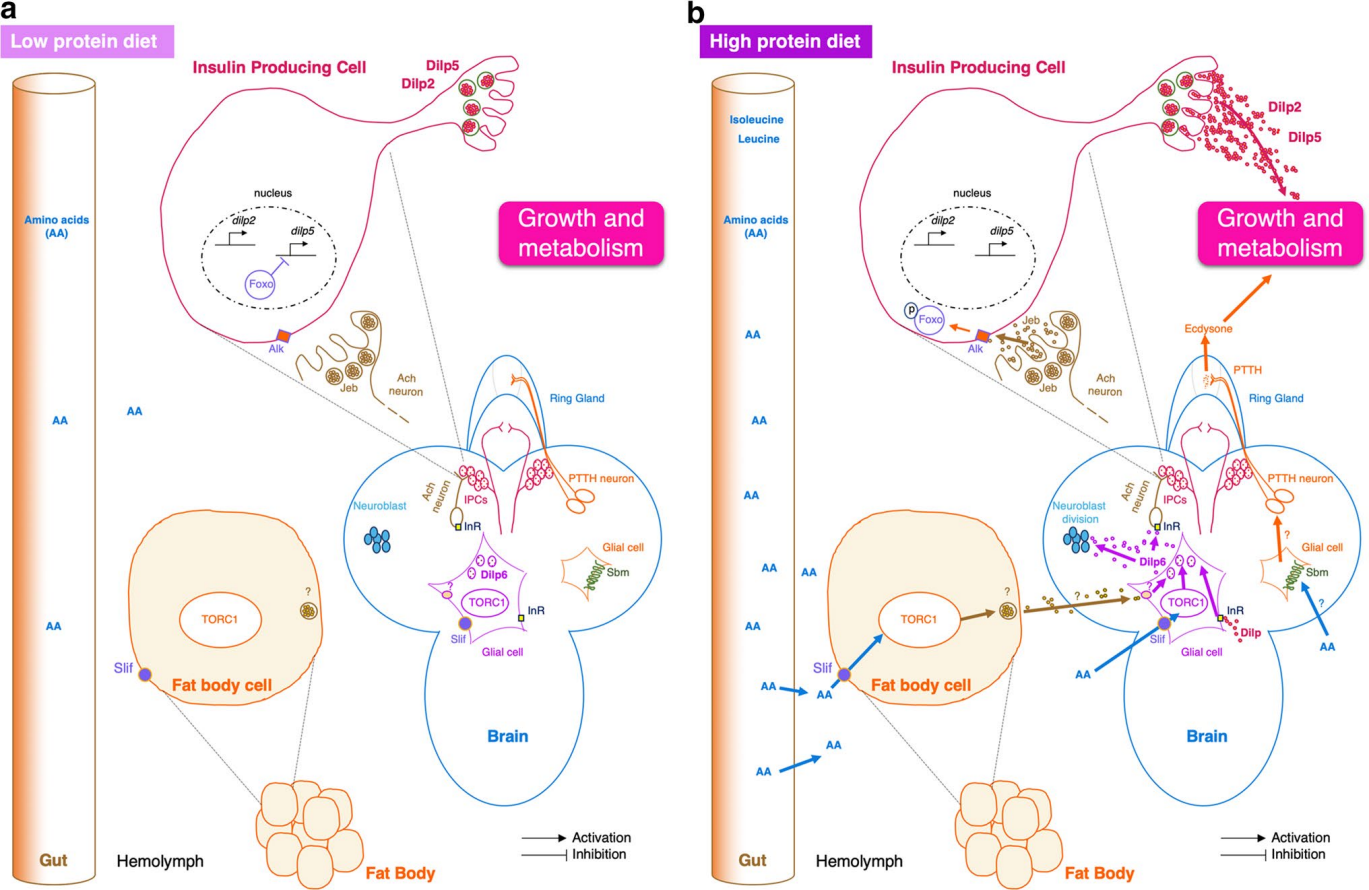
Amino acid sensing by glial cells. Glial cells detect amino acids to control metabolism and growth. Comparison of low- (**a**) and high-protein diet (**b**) situations shows that in a high-protein diet, amino acids activate the secretion of an unknown factor by the fat body that induces the release of DILP6 by glial cells. Furthermore, glial cells can directly detect amino acids by TORC1 via the Slif amino acid transporter and secrete DILP6. DILP6 promotes the neuroblast reactivation and the release of Jelly belly (Jeb) by acetylcholinergic neurons. Jeb promotes the FoxO phosphorylation in IPCs to allow the *dilp5* gene expression and DILPs release. Glial cells express another amino acid transporter Sobremesa (Sbm). Sobremesa is involved in larval development by regulating PTTH secretion and therefore ecdysone. *AA* amino acids, *Ach neuron* acetylcholinergic neurons, *Alk* anaplastic lymphoma kinase receptor, *IPCs* insulin producing cells, *InR* insulin receptor, *Jeb* Jelly belly, *PTTH* prothoracicotropic hormone, *Sbm* Sobremesa, *Slif* Slimfast, *TORC1* target of rapamycin

In the brain, glial cells are also able to detect amino acids and regulate cell growth and larval development (Fig. 5). They can directly react to amino acids transported by the amino acid transporter, Slif. In response to amino acids, glial cells release an insulin-like peptide, DILP6. Then, DILP6 binds to InR located on some acetylcholinergic neurons in the brain. This neuronal activation allows the release of the Jelly belly (Jeb) peptide (Fig. 5) [43]. The mechanism of Jeb secretion is unknown but is different from Acetylcholine release. Jeb peptide binds to Alk (Anaplastic lymphoma kinase receptor) expressed by IPCs. It allows the activation of a PI3K pathway that leads to the phosphorylation of the transcription factor FoxO. The phosphorylated inactive form of FoxO remains in the cytoplasm allowing the expression of *dilp5*. Finally, the secretion of DILP5 into the hemolymph promotes larval growth (Fig. 5). In low protein diet condition, the transcription factor FoxO represses the expression of *dilp5* gene in IPCs. Some glial cells expressing the InR receptor are sensitive to DILP5 and other DILPs and express *dilp6* generating a feedback loop (Fig. 5) [43].

Larval development coordination involves also many hormones including the prothoracicotropic hormone (PTTH) and ecdysone, a steroidogenic hormone [44]. These hormones must be finely regulated to control the duration of the different larval stages and the transition to pupal stage. Before each larval or pupal molt, the increase of PTTH synthesis and secretion by PTTH neurons located in the larval brain activates ecdysone secretion by cells localized in the Ring Gland (Fig. 5). This ecdysone production may also depend on the presence of various nutrients such as amino acids since it is under the control of DILPs [45]. Moreover, it has been shown that the amino acid transporter coding gene Sobremesa (Sbm) expressed in glial cells is involved in the timing of larval and brain development [46]. Sbm downregulation causes an extension of the duration of the last larval instar together with an increased body size, but leads to smaller brain lobes. Downregulation of Sbm in glia cells does not affect *dilp6* expression in glia but reduces PTTH levels (Fig. 5) [46]. This drop of PTTH might lead to a decrease of ecdysone synthesis inducing a developmental delay and a reduction of the brain size due to less neuroblast division [47]. The mechanism of how glial cells govern PTTH level in PTTH neurons remains elusive. In glial cells, Sbm could participate to the transport of circulating amino acids in hemolymph to promote PTTH release necessary for ecdysone synthesis required to regulate developmental timing [46].

During the *Drosophila* larval development, some cells like neuroblasts remain quiescent in the brain until they enter cell division to generate two cell types, neurons and glial cells [48]. The activation of neuroblast division depends on amino acids present in the food; whereas, differentiation of neuroblasts into neurons and/or glial cells is food independent [24, 25, 49]. The fat body senses the amino acids via the TORC1 pathway and produces an unknown signal. This signal acts on brain glia cells which in turn release DILP6 [24, 25]. DILP6 binds to InR located on neuroblast membranes, inducing neuroblast reactivation and division (Fig. 5) [24, 25].

## Conclusion and outlook

Amino acids are essential as basic components for protein synthesis that are involved in many cellular functions (membrane activities, enzymes, transports, signaling, and gene expression). Amino acids can also act as neurotransmitters like glutamate and participate as precursors in the synthesis of neurotransmitters (tryptophan for serotonin or phenylalanine for dopamine). Some amino acids like glutamate can be synthesized by the cellular enzymatic machinery. The so-called essential amino acids like tryptophan, phenylalanine or leucine are not synthesized by the organism, and must absolutely be found in the food. Thus, among the nutrients capable of regulating larval growth and development, amino acids play a major role.

Here, we highlight known internal detection mechanisms and pathways depending on amino acids that regulate larval and tissue growth. Once in larval hemolymph, circulating amino acids are mainly detected by the fat body, and by neurons and glial cells in the brain. This detection leads to the release of various factors, which allow the coordination of larval systemic growth. Usually to study the role of amino acids in *Drosophila*, a mix of amino acids like yeast or peptone extracts are used as a source of amino acids [35, 50]. Few essential amino acids have been shown to play a key role in development or in food intake [31, 40, 42].

Other cells than the specific neurons and glial cell we described above are also sensitive to amino acids in larvae. For example, the fat body cells are sensitive to these nutrients, which in turn can influence larval growth. In this regard, during mid-third instar Class IV multidendritic nociceptive ppk peripheral neurons react to the loss of environmental arginine via Slif. Then, they activate glutamatergic neurons in the ventral chord, which in turn stimulate Dilp2 release by IPCs [51, 52]. Imaginal discs seem also to be sensitive to the presence of nutrients including amino acids via the activity of the tumor suppressor PTEN (phosphatase and tensin homolog deleted on chromosome 10). This effect of nutrients on imaginal discs can potentially influence their growth and proliferation in specific conditions [34].

In *Drosophila* adults, nutrients and amino acids are also crucial since flies need food resources to regulate their life cycle, to survive, to have social interactions and to reproduce. In adults, the essential amino acid threonine promotes sleep via GABA neurons in the brain and three other amino acids (l-Glutamate, l-Alanine and l-Aspartate) are able to directly stimulate DH44 + brain neurons to increase in food consumption via a putative amino acid transporter, CG13248 [53, 54]. In adult females, nutrients play also an important role in germline stem cells development. This requires specific nutrient-responsive signaling pathways which includes insulin/IGF signaling, TOR signaling and GCN2-dependent amino sensing [55]. This indicates that similar molecular pathways can be mobilized all along *Drosophila* life cycle to sense amino acids in different cell types.

In mammals, amino acids are detected by the digestive tract to release gut hormones like cholecystokinin hormone (CCK) to regulate food intake. Surprisingly, in *Drosophila*, such mechanism of detection of amino acids by the gut to regulate DILPs or food intake has not been discovered yet. However, a recent study highlights the presence of intestinal cells capable to detect amino acids but the role of these cells remains unknown [56]. Further characterizations and analyzes are necessary to understand the exact role of amino acids on larval development and growth.
